# How Do Migrant Nursing Home Staff Relate to Religion in Their Work With Patients Who Are Approaching Death?

**DOI:** 10.1177/0898010120973544

**Published:** 2020-11-25

**Authors:** Marta Høyland Lavik, Birgitta Haga Gripsrud, Ellen Ramvi

**Affiliations:** Chaplaincy Department, Stavanger University Hospital, Norway; Stellenbosch University, South Africa; Department of Caring and Ethics, Faculty of Health Sciences, University of Stavanger, Norway; Department of Caring and Ethics, Faculty of Health Sciences, University of Stavanger, Norway

**Keywords:** religion, holistic nursing, migrant health care workers, death

## Abstract

**Aim:**

To investigate how migrant nursing home staff relate to religion in their care for patients who are approaching death.

**Method and Theory:**

Individual in-depth interviews were conducted with 16 migrant health care workers from five nursing homes in Norway. The overall analytic approach was hermeneutical. The parts and the whole were interpreted in light of each other to gain a “thick description” of the data material in order to show the ways in which experiential meaning-making draws on cultural webs of sign ificance.

**Findings:**

Religion held various meanings for the migrant health care workers interviewed. Religious and cultural competence and knowledge of migrant nursing home staff was neither asked for by the management nor discussed in the staff group. The way our participants related to religion at work was therefore based on individual preferences and internalized practices.

**Conclusion and Implication for Practice:**

Organized reflection groups among staff are needed in order to integrate and develop religious literacy in the multicultural nursing home setting. Such reflection groups can help the individual staff member to perform holistic nursing, that is, to be attentive of the interconnectedness of biological, social, psychosocial, and spiritual aspects in a human being.

## Introduction

Of all persons who die in Norway, 51% die in a nursing home, and health care workers in nursing homes are thus continuously confronted with death at work (The Norwegian Institute of Public Health, 2018). Due to globalization and migration in an increasingly interconnected world, a larger proportion of health care workers in Norway today have a migrant background.^1^ In 2017, more than 17% of the work force in Norwegian nursing homes were migrants, whereas the majority of the patients were still ethnic Norwegian (Claus, 2018). The number of staff from African and Eastern European countries has increased over the past decade in municipal care whereas staff from Nordic countries has decreased, correspondingly. A third of the full-time equivalent in municipal care in 2017 was delivered by staff of Asian background (Claus, 2018). Transnational migration has been defined as “a process of movement and settlement across international borders in which individuals maintain or build multiple networks of connection to their country of origin while at the same time settling in a new country” (Fouron & Glick-Schiller, 2001 p. 60). Transnational migration also highlights a relationship between high-income countries’ need for cheap labor and the availability of such labor in low-income countries due to lack of opportunities, poverty, and scarcity of resources (Kittay et al., 2005, pp. 449-452; Loftsdóttir & Jensen, 2016; Sørensen et al., 2008; Tingvold & Fagertun 2020; United Nations, 2017).

## Aim

Little is known about how the influx of migrant labor into Norwegian nursing homes has affected care practices (cf. Munkejord, 2016). This article is based on in-depth interviews with 16 migrant health care workers about their encounters with dying and death in Norwegian nursing homes. The participants were born in countries where family-based care is the most common care practice, and where religion for the most part is seen as an integral part of making sense of reality at both societal and individual levels. Having migrated to Norway and been employed as nurses and ancillary staff in nursing homes, our participants find themselves in a more secularized country than their country of origin, and in a country where care for the dying for the most part is provided by institutions like nursing homes and hospitals (Thoresen, 2017, pp. 276-277). The complexity represented by this context made us wonder: How do migrant nurses and nurse assistants relate to religion in their work with patients who are approaching death in nursing homes in Norway?

## Background and Significance

In order to develop a background for our data interpretation, we will first define the umbrella term *spirituality*. Following on from this, we will attend to the lines of secularization and multireligiosity in the face of death. Last, we will touch upon how historically, in this national context, religiosity, nursing, and health have been interconnected.

### The Umbrella Term *Spirituality*

The definition of spirituality has developed and expanded over the years from being applied only within religious traditions about experiences of the sacred, to becoming a term differentiated from religious institutions and authorities. The word *spirituality* comes from the Latin noun *spiritus*, which means breath, wind, or ghost/spirit (https://www.naob.no/ordbok/spiritus). In early Christianity, spirituality was defined narrowly to describe a life oriented toward the Holy Spirit. In the Middle Ages, the term was expanded to also include mental aspects of life (https://en.wikipedia.org/wiki/Spirituality). Today, spirituality has become somewhat detached from its religious roots, and is applied more broadly about a range of subjective human experiences of meaningfulness, such as deeper values, existential, and/or religious topics (cf. Koenig et al., 2012, pp. 37-38; Paul Victor & Treschuk, 2020, p. 108; and the definition of spiritual care by European Association for Palliative Care, n.d.). In this article, the word *spirituality* is thus used accordingly as an umbrella term to cover these three areas: (1) the existential, (2) the value-based, and/or (3) the religious. Spiritual issues include questions or issues of one, two, or all of these three areas, encompassing the answers, solutions, strategies, thoughts, and feelings a human being would rely on in order to manage such issues. This broad definition of the term suggests all human beings *qua* human beings to be spiritual creatures (Hawthorne & Gordon, 2020, p. 147).^2^ Not all people have a religious belief, but human beings per se—consciously or unconsciously—have a view of life and a set of values to navigate after. Spiritual issues do not necessarily evoke only positive feelings in a human being, as emphasized by the American psychologist, Kenneth I. Pargament. He states, “Spirituality is fully interwoven into human experience [. . .]” and is “a potential resource as well as a potential source of problems” (Pargament, 2007, pp. 343-344). Negative effects of spirituality can for instance be caused by an individual’s inner religious struggle, by religious groups discouraging certain forms of medical care, or by physical and/or sexual abuse (Oman & Thoresen, 2005, p. 452). Although there is a considerable body of research indicating religious involvement to be favorable to health (see Doka, 2011, pp. 101-103; Koenig et al., 2012, pp. 94-120; Page et al., 2020, pp. 91-95, for numerous references), there are also studies that reveal how religious involvement can undermine psychological well-being. The latter can be seen, for instance, when people experience marital problems, challenging parent–child relations, or problematic interactions with fellow church members or clergy (for references, see Doka, 2011, pp. 102-103; Koenig et al., 2012, pp. 53-93; Page et al., 2020, p. 95).

Although spirituality remains a useful umbrella concept in our context, many of our participants clearly identify with a religious tradition, and implicitly or explicitly refer to dimensions of religion such as religious affiliation, religious rituals, and religious coping strategies. For this reason, we will mainly be relying on the concept of religion rather than spirituality in our interpretation of the data material. Religion comes from the Latin word *religare. Re* means “back,” and *ligare* means “bind together,” and the meaning of the word *religion* is therefore literally “to bind back together” (Koenig et al., 2012, p. 605). In this article, religion is therefore defined as yet another multidimensional concept that often, but not always, involves an organized system of beliefs, behaviors, practices, rituals, and ceremonies (conducted privately or in public) related to what is often described as the mystical, the sacred, the transcended (cf. Koenig et al., 2012, pp. 37, 45; Page et al., 2020, p. 90; Paul Victor & Treschuk, 2020, pp. 109-110).

### Secularization and Multireligiosity in the Face of Death

Our participants were born and raised in societies that tilt more toward the religious than the secularized. Globally, practices around death are formed and affected by a range of sociological factors:

As in life, so in death: we find global patterns, and we find both enduring and emerging variations, and these variations are by nation as well as by the more conventional sociological variables of gender, class, ethnicity and religion. (Walter, 2012, p. 139)

The religious versus the secularized in Norway can be described and interpreted in a variety of ways as secularization (as well as religion) is a multidimensional concept (cf. Botvar, 2010). However, at least two developmental lines may be traced. Although Norway had a state church until 2017, a 500–year long Protestant tradition has gradually made religious affiliation a mainly private, and not a public, issue.^3^ Secularization tends to be characterized by *differentiation* (separation of state and church), *diminishing religious faith and practices* (decreased religious conviction, decreased participation in religious services, and ceremonial rituals like baptizing and marriage), as well as the *increasing* *privateness* *of religious practice* (decreased participation in organized religion, e.g., as members of the Church of Norway; Horsfjord et al., 2018, p. 282; Taule, 2014, p. 10). This means that meaning-making in Norway—both on a societal level and on an individual level—gradually has been more and more detached from religious norms and authorities (cf. Schmidt, 2010b, pp. 196-204). The same applies to public welfare institutions such as nursing homes. Religion is not used as an interpretative tool in making sense of experiences or incidents in the nursing home.^4^ Along with the secularization process, however, Norway has seen migration over the past 50 years, and migration takes the nation in a more multireligious direction (Schmidt, 2010a, p. 32).^5^ The long-term outcomes and consequences of these two developmental lines, existing side by side, are difficult to predict, but a multifaceted intertwinement of the religious and the secular is proposed to exist in contemporary societies (Schmidt, 2010a, pp. 25-43; Wojtkowiak, 2018, p. 464). These broad developmental lines make an important background for understanding the present study.

In addition to navigating between the religious and the secularized, our participants traverse the private and the institutionalized arenas in their care for the dying as all of them come from countries where this care is family-based (cf. Ådland et al. 2020; Egede-Nissen et al., 2017). How death is understood in the nursing home in Norway is informed by parallel developments surrounding death in the Nordic societies at large (cf. Høeg & Pajari, 2013, pp. 109-115; Thoresen, 2017, pp. 269-270). Beyond the French historian Philippe Ariès’s well-known four phases in humankind’s understanding of death (Ariès, 1974) is the Danish sociologist Michael Hviid Jacobsen’s (2016) notion of a fifth phase, the spectacular death. In his view, the adjective spectacular is applied about death as “something that we witness at a safe distance with equal amounts of fascination and abhorrence, we wallow in it and want to know about it without getting too close to it” (p. 10). The Norwegian theologian Eivor Andersen Oftestad proposes yet another phase, the individualized or autonomous death, which may not sequentially succeed but at least lives side by side with the idea of the spectacular death (Oftestad, 2019). In this phase, which is under development, it seems as if only the imagination can put a limit to what individuals can decide about the orchestration of their own death.

In the nursing home, death presents itself as quite ordinary and conventionally regulated in comparison with these recent cultural developmental lines. Still, death in the nursing home does not happen in a vacuum, and our participants continuously negotiate between the institutionalized and the private as well as between the secularized and the religious in their care for the dying.

### The Interconnectedness of Religiosity, Nursing, and Health

As part of the contextual backdrop of the present study, it is worth noting the historical evolution of the nursing profession and the understanding of spiritual care as integral to the nursing act. Historically, there has been an affinity between religion and nursing. As long back as in Medieval times, religious organizations and monasteries took care of the sick, and were the predecessors for the modern hospital (Page et al., 2020, p. 90). A fundamental principle of the nursing profession is to care for the whole human being, and up to the early part of the 20th century, nursing students were taught to care for the patient’s spirit, mind, and body (Page et al., 2020, p. 91). This holistic caring principle grew out of the biblical parable of The Good Samaritan to be found in Luke 10:25-37 (Martinsen, 2017, pp. 31-32). The first professional nurses in Norway were educated in private diaconal institutions in the capital Oslo from 1868 onward, and before this in Copenhagen from 1863 (Martinsen, 2017, p. 20). This diaconal nursing education was rooted in Germany, where women were educated to relieve social and spiritual suffering, and to alleviate illness through voluntary work organized by the church. Diaconal nursing emerged from a Christian tradition, based on biblical-religious thinking—a context quite different to today’s nursing education, theory, and practice—although diaconal health education institutions still exist alongside the state’s higher education institutions. With the arrival of modern medicine, the relationship between nursing and religiosity became less visible, and “care shifted from the care for the spirit, mind and body to caring for the biopsychosocial being” (Page et al., 2020, p. 91). Aspects of the religious values and understandings associated with diaconal nursing do, however, remain relevant to the universal foundation of nursing today (e.g., patient-centeredness, professional judgment, moral capability; cf. Martinsen, 2017).

Health care workers in Norway are obliged to provide for physical, psychosocial, and spiritual needs of the patient (cf. Giske & Cone, 2015, p. 2927; Medås et al., 2017, p. 274).^6^ This national legislation is anchored in the United Nations’ (1948) Universal Declaration of Human Rights, Article 18:

Everyone has the right to freedom of thought, conscience and religion; this right includes freedom to change his religion or belief, and freedom, either alone or in community with others and in public or private, to manifest his religion or belief in teaching, practice, worship and observance (https://www.un.org/en/universal-declaration-human-rights/).

The obligation is also anchored in The World Health Organization’s (2020) “Majority provider of palliative and end-of-life care services in community for dementia [public, private]” statement: Ill and dying should receive care

[. . .] through the prevention and relief of suffering by means of early identification and impeccable assessment and treatment of pain and other problems, physical, psychosocial and spiritual.

In order to perform integrated, holistic, or comprehensive care, Norwegian official documents describe this as attending to and providing for patients’ physical, psychosocial, and spiritual needs.^7^ In the national academic guidelines for nurses who care for dying people, it is emphasized that communication with dying patients should, among other topics, be about the emotional conditions, including existential issues (The Norwegian Directorate of Health, 2019, p. 18). However, according to the guidelines there are indications that such issues are currently not well enough catered for in Norwegian health care services (The Norwegian Directorate of Health, 2019, pp. 34-35, pp. 37-42).

However, our focus in this article is not on the patient but on the migrant nursing home staff and how they relate to religion in their work with patients who are approaching death.

## Research Design

This study is part of a major research project (MultiCare) that examines the multicultural staff community in Norwegian nursing homes, and how labor might be an arena of integration and inclusion.

### Method

As part of our Work Package in this project, individual in-depth interviews were conducted between September and November 2017 with migrant nurses and nursing assistants in five different nursing homes in the western part of Norway, 13 women and three men.^8^ The participants in the study were aged between 26 and 55 at the time of the interview, and had migrated to Norway from nine countries in Southeast Asia, East Africa, and Eastern Europe. Thirteen of the participants had a Christian affiliation (Orthodox, Catholic, Protestant), whereas two had a Buddhist upbringing, and one a Muslim upbringing. The researchers hoped for more variation in terms of religious backgrounds but still regard the interview material as rich and heterogeneous despite the somewhat homogeneous religious affiliation.

Most participants had lived in Norway less than 10 years at the time of the interview. They came to Norway as refugees, asylum seekers, labor migrants, or marital migrants. Three had been certified as nurses in their country of origin, but their education was not approved in the Norwegian system. At the time of the interviews, six worked as nurses and 10 as nursing assistants in five different nursing homes. Five of the participants had some sort of work experience from their country of origin.

Participants were recruited through both written and verbal requests channeled through the nursing homes’ management teams. They, in turn, contacted their respective line managers who received written information about the project. The line managers passed on the written information to staff who were born outside Western Europe. The enrolment criteria were health care workers (nurses and ancillary staff) who were born and raised in countries outside the Western European, and who had come to Norway as labor migrants, refugees, marital migrants, or other. Those who were approached and were interested to know more about the project gave the line manager permission to give their telephone number to the researchers. The first and last author contacted those interested, gave more verbal information, and made appointments for an interview. Throughout the study, we emphasized the ethical principles of respect, protection from harm, informed and voluntary consent, anonymity, and proper data storage (The Norwegian Research Ethics Committees, 2014). Informed consent was obtained from all participants, and they were assured that they could withdraw from the study at any time without risking any consequences.

In-depth interviews took place in a suitable undisturbed room at the nursing home during the participant’s work time, with permission granted by the nursing home’s management team. The interviews were conducted in Norwegian, and some language difficulties occasionally occurred. At times the participants struggled to find the right words to describe something, and the language sometimes became oversimplified. In such instances the researcher asked the participant to repeat or clarify to make sure the researcher had comprehended what was communicated. The participant also sometimes asked for clarification if the question from the researcher was imprecise, and the researcher rephrased it until a common understanding was attained. The first and last authors conducted the first two interviews together. Subsequent interviews were divided between the two authors (7 interviews each, *n* = 16). The interviews were recorded and afterwards transcribed verbatim by the two authors who conducted the data collection. Each interview lasted approximately 1½ hours. To protect their identities, all names used for participants in this article are fictitious.

The overall research problem of the Work Package was to investigate the extent to which migrants working as health care workers in nursing homes in Norway experienced their cultural and religious background as relevant in the course of a patient’s death. The interviews were thus guided correspondingly by a semistructured interview guide consisting of three parts: (1) What experiences did participants have with death from their home country? (2) How do participants experience working with dying patients/death in a Norwegian nursing home? (3) How do participants feel their work with dying patients/death affects them—personally and professionally? Where appropriate, researchers asked the participants to give concrete examples and stories to illustrate their statements.

### Theory

Interpretation is not only a characteristic activity of human beings as language-driven and communicative creatures but also a universally constitutive factor of human behavior and our search for understanding through meaning-making (Gadamer, 2011; Sørensen et al., 2008, p. 103). Experiential meaning-making is in many ways a psychosocial phenomenon in which inner (intra- and inter-psychic) and outer (sociocultural, societal, and institutional) worlds combine and feed into each other. Hence, with these aspects of intertwining human experience and culture in mind (onto-epistemologically), our overall analytic approach is hermeneutical. This article aims at interpreting the parts and the whole data set in light of each other in order to gain a “thick description” of the material and the ways in which experiential meaning-making draws on cultural webs of significance (Geertz, 2017, pp. 3-33). The hermeneutic operation is such that an interpretation of details may well affect the interpretation of the whole, and vice versa. This process of interpretation is known as the hermeneutical circle. According to the German philosopher Hans-Georg Gadamer, being aware of one’s own preunderstandings and prejudices is vital in the act of interpreting as prejudice and understanding are intertwined in the sense that prejudices are present in all understanding. This is again connected to Gadamer’s (2011) concept of horizons of understanding. In every encounter between human beings, different horizons of understanding meet:

In fact the horizon of the present is continually in the process of being formed because we are continually having to test all our prejudices. An important part of this testing occurs in encountering the past and in understanding the tradition from which we come. Hence the horizon of the present cannot be formed without the past. [. . .] *understanding is always the fusion of these horizons supposedly existing by themselves*. (p. 305)^9^

For the authors of the present article, the process of analyzing the data became a reminder of how complex Gadamer’s (2011) idea of the fusion of horizons is, but at the same time it revealed how rewarding a process of understanding can be when preunderstandings and prejudices are sought out, identified, and understood. For us, it was especially the notion of religion (a resource, a problem, both, or none of these) that was discussed throughout the whole process (cf. Jong, 2014). Through our interdisciplinary discussions, our various preunderstandings and prejudices have materialized themselves as significant perceptions and insights to be discussed in relation to the data material, revealing our respective blind spots. We have actively relied on differences in our personal and professional backgrounds to develop a thick description and (through interpretation) gain a thick understanding of the data set and ourselves. In our effort to reveal our own prejudices we have experienced not a fusion of horizons but perhaps more a continuing fusing of horizons—understood as a never-ending dynamic process of understanding (Elness-Hanson, 2017, p. 36).

Hermeneutical research includes various approaches, and in this article it was combined with the method of thematic analysis in an early stage of attempting to sort and systematize the data. Thematic analysis aims at identifying and categorizing essential themes that form the data (Braun & Clarke, 2006). The essential themes which have emerged in this process have assisted us in carrying out a further hermeneutic exploration of the research question: How do migrant nursing home staff relate to religion in their work with patients who are approaching death?

## Findings

From a previous study conducted on the same data set, authors concluded that health care workers who work in nursing homes and are confronted with death at work experience situations that can activate in them questions of existential character (Ramvi & Lavik, 2019). In the present study, we wanted to investigate the extent to which migrant health care workers in nursing homes in Norway experienced their cultural and religious background as relevant in the course of a patient’s death. In all interviews it became clear that none of our participants had experienced the management in the nursing home to ask them about their cultural and religious background. Our strong impression is nevertheless that the health care workers relate to their backgrounds in various ways. The main theme that emerges from our data material shows the wide span of health care workers’ experiences of religion in their care for patients who are approaching death. This main theme is explicated in three subthemes, which will be presented in the following section.

### The Wide Span of Health Care Workers’ Experiences of Religion

All 16 participants come from societies that are less secularized than the Norwegian, and they tell us how important religion is in the country of origin with regard to the view of death (e.g., fear of death) and treatment of the dead. We were therefore curious to find out how they would relate to religion in their care for patients who are approaching death in Norwegian nursing homes.

a. “I prayed to God that I should be strong and help him.”—religion as a source of comfort and strength in the health care worker

In Norway, end-of-life care is welfare-based and often carried out in institutions, which means that health care workers in many cases spend more time around the patient than family members are able to do. The exception is when a patient is dying. Then, the family can be present in the patient room day and night should they wish to. This way of organizing the care is very different from what our participants are used to from their countries of origin. It was, for instance, a shock for Isabella, a 30-year-old woman from East Africa, when she as a young woman, and new to Norway, worked as an assistant in a nursing home and became aware that the son of a patient did not want to be with his father who was dying. For her, this was an unfamiliar way of relating to dying:

One man was very ill. I was together with another nurse, and she cared for him and she said: “He is going to die now.” “Oh?!” [participant flung her arms out]. I trembled and ran out and started crying. I said: “No, that cannot happen!” My colleague said: “Isabella, he is here, he is going to die”. “Doesn’t he have a family?” I asked. “Yes, he has a son, but he does not want to be here.” Then I started crying even more. [. . .] Had it been now I would have accepted it, because now I know a little more about Norwegian culture, but this was in 2005. [. . .] I just sat like this [face in her hands], and just cried. I felt quite faint. I was concerned about he who died, I was afraid of thinking of how it would be for the son. “Is it like this in Norway?” I was completely shaken up. It was so contrary to what I was familiar with. I had not accepted . . . OK, then after that, when we were finished caring for him, I went to another room and prayed to God that I should be strong and [able to] help him [the son].

Initially, Isabella felt troubled, distressed, and upset when she realized how end-of-life care could be carried out in Norway. However, she seems to have gone through a cultural hardening or acclimatization—although she reacted strongly at first, she then states that at the current time, she would accept this manner of behavior. In order to digest the difficulties she experiences in this specific situation, Isabella removes herself from it and prays to God. She identifies with an Orthodox Christianity of Eastern Africa. In our interpretation of her narrative, she uses her religion on several levels—as a ritual to give her some sense of self-relief, and as a source of seeking comfort and strength in the familiar and recognizable in a situation she experienced as highly unfamiliar, upsetting, and frightening and experienced an embodied reaction to.

b. “It was lovely to hear that we can sing together.”—religion as a means of cross-cultural sharing between patient and health care worker

Grace is a 28-year-old woman from Southeast Asia. She identifies herself as a Roman Catholic. She tells us how surprised she was when she arrived in Norway and realized there are factors other than religious that can be constitutive in peoples’ meaning-making:

When I first came to Norway and heard that some do not believe in God [I thought]: “What?!” I was like: “How can they live without God? He who gives us life?” It was very strange to think of for me. In [my country of origin] Christianity is the religion most people belong to, 80% perhaps are Catholics. It was a completely new idea that there could be many people in Norway who do not believe in God.

Grace seems to draw on her own internalized religion in her encounters with the patients. She gives two examples of this, first an example of what she can say to the patients:

[. . . for example,] when I am on my way out after work [I can say to patients]: “I will pray for you, I will think of you. And I hope you will sleep well,” and such things. “Thank you, I appreciate that,” they [the patients] then reply.

Then Grace gives an example of how she connects with the patients through singing:

I begin singing, and often they [patients] say: “Oh, do you know that one? Yes, I have a hymnbook, too,” and then they start singing together with me, and “It was lovely to hear that we can sing together. It was so lovely!” [the patients say].

According to Grace’ narrative, the patients are somewhat surprised to find that she knows the same hymns as them, despite their cultural differences and age differences. Singing hymns they both know allows them to share something of experienced value interculturally, and this is to be cherished, not only for the patients but presumably also for Grace. This partaking in a common ground of Christian tradition displays how a communal fellowship can be created despite different age and culture, and apparently it produces a sense of meaningfulness, closeness, and familiarity in Grace.

Eva, 55 years old, from another country in Southeast Asia, converted from Buddhism to Christianity (Roman Catholicism) as an adult. She sometimes uses the Bible to bring words to a person who is dying and no longer able to talk:

Researcher: When you sit with somebody who is dying and you hold their hand, what do you talk about—if the person is able to talk?

Eva: Often they are unable to talk. Sometimes I read something from the Bible for them, until they let go of my hand, then I leave them. But before I leave them I go close and say: “I’ll be back in 15 minutes. You are not alone.” They should not be alone.

Eva does not say which passages she reads from the Bible, but as far as we understand, she uses the Bible and herself to provide fellowship and presence to the dying patient. She does this through the tactile and the audible, through holding the patient’s hand, and through reading from the Bible. Also for Eva, the Bible is a common ground of cross-cultural sharing between her and the patient—a way of being together.

Philippa, 35 years old, from a country in East-Africa, has a different approach to Grace and Eva. Philippa wears a hijab, and this symbolic marker makes her Muslim religious affiliation visible to others. She works in an environment where most of the patients are ethnic Norwegians and do not share her Muslim faith, and she says the following about how to enter into the patients’ religious sphere:

I cannot start here with my faith or my culture. They have their own culture and faith, the old. You are to follow the things they have. First and foremost it is respect which is important. We must have respect for all religions, whether it is a Jew, Christian, Islam, Hinduism or Buddhism, it is sort of . . . everyone has their own faith, and you have to respect the one they have. It is not like “Oh, she has *that* culture, and then you start . . .,” no it is not like this. I have to give respect, and help the person who needs help from me.

For Philippa the question seems to be how to live with religious differences rather than similarity or shared Christian religion (as for Eva and Grace). She accepts the elderly as having their own faith and culture. The key to dealing with this as a professional, according to her, is to give respect, in providing the help that is needed regardless of the patient’s or her own faith. Philippa’s experience of having her own religious faith seems to produce respect for the patients’ potential religious faith. The way she reflects upon her own religious faith in relation to the patients’ individual needs is in accordance with the official Norwegian guidelines: The professionals are to provide for the patient’s physical, psychosocial, and spiritual needs.

Isabella, whom we presented above, has experienced many times that the patients are interested in religious issues, and she provides for their spiritual needs:

[The patient asks her]: “Isabella, can you pray for me, can you read the Bible?” “Yes, I can read, which Psalm would you like?” And I read for her. It was she who asked me. We have also had a lady who reads every morning. And she can say: “Oh, is it you?” and I read to her—she couldn’t see very well. But whether they [the patients] are spiritual or not, I pray inside. Whether it is allowed or not allowed, I do it in my heart. Regardless.

Isabella uses her own internalized religious faith and prays for the patients. Regardless of how the patients may feel about religion, she will pray for them inside, in her heart. This ritual of praying within seems to be an integral part of her way of practicing her religion. As they are neither audible nor visible, she takes the chance to perform her prayers at work.

c. “I have never ever heard that religion is important.”—religious rituals as part of the life cycle?

Odette is a 30-year-old woman from Eastern Europe who identifies herself as a Roman Catholic. More than 90% of the inhabitants in her home country belong to the Roman Catholic Church, and she explains the church as a central institution in peoples’ life cycle. For Odette’s elderly grandmother the family follow a common practice of the Roman Catholic Church:

In [my country of origin] many are involved with the church. My grandmother is not very involved with the church, but we have asked the priest to go and see her once a month. He visits her in her home so that she can stay in touch with religion now as she is no longer able to go to church.

When Odette compares Norway with her home country in terms of church engagement in old age, she has the clear impression that most nursing home patients in Norway seem not to find church and religion relevant when death is approaching:

When you ask questions [to the patient]: “What is important to you now?” Some are very involved with the family, some are interested in food, so they are different, but that religion is important, I have never heard. Since I started worked in nursing home NN [the former], I have *never* ever heard that religion is important. *Never*.

Odette is surprised to observe this lack of church engagement in the later stages of life, and she sees a contrast when she compares Norway with her country of origin. In her Roman Catholic tradition every member has a duty to attend church, and when sickness in old age prevents the individual from doing so, the priest can pay home visits. This is done to let the dying person confess sins and receive the sacraments of the Eucharist and of the Anointing of the Sick. When Odette now works in a nursing home in Norway, and registers that people are not calling for the priest, this stands in sharp contrast to what she is used to from her country of origin where

[. . .] most elderly people ask the priest to come. And they want to confess their sins. They want to talk. Sometimes, if they are in the terminal phase, they tell their whole life story, and they confess the sins they have done throughout their lifespan. Then they ask for forgiveness. And this I have *never* seen here!

Charlotte, a 38-year-old from another Eastern European country, also identifies as a Roman Catholic. She refers to her internalized religious faith as something troubling. She is for instance wondering why she has changed when it comes to church attendance after arriving in Norway:

Charlotte: [. . .] when I lived in my home country I was active [in the church], but when I moved to Norway I am not like that. We went to church every Sunday earlier, but now I work and I find it is slightly different. I don’t know why it is like that [laughs a little]. It is not good. No, it is not good.

Researcher: What causes this change, do you think?

Charlotte: [. . .] I had more time, but it was also more compulsory—we go to church—but here the Sunday comes and I work and the children don’t consider going to church, and neither does my husband [laughs].

Having moved to Norway—where church attendance is not compulsory in the majority religion, Lutheran Christianity—she seems to feel some sense of guilt for not keeping up regular weekly attendance: “It is not good,” she says twice. At the same time, this quote lets it shine through that Charlotte can allow herself and her family to loosen the ties to the church now as she is in a different sociocultural setting.

Another participant who has loosened the ties to her traditional religious conviction is Eva, as mentioned earlier. She recalls being very scared as a child when her mother with a Buddhist faith told her that people who died became ghosts who could come and visit the living. She still thinks of the ghosts sometimes but assures her mother she is not scared anymore:

Now we are in Norway. I don’t think there are any ghosts here [laughs a little]. When I call my mother I can say: “Mum, I am in Norway, so there are no ghosts here, and they do not talk about it either.” “That is good,” my mum says.

After having moved to a different sociocultural setting, Eva experiences an alternative horizon of understanding, to use Gadamer’s (2011) words. The displeasing memories from the tradition of her upbringing are not forgotten, but have faded, and are replaced by a confidence that there are no ghosts which can visit the living.

If we go back to Charlotte, she reveals how her Roman Catholic religious tradition unsettles her. When the researcher asks her if she sometimes thinks of her own death as she works so close to people who approach death, she replies “no” and returns to the question of religion:

No. [laughs again], but this I can say, I sometimes think that I perhaps have to start living a little better. I want to go to heaven when I die. That is the Catholic religion. Perhaps I think I have to revert my life, and live better or become better. I think a little like this. It is the Catholic religion. Everyone wants to come to heaven to God.

Charlotte’s upbringing within the Catholic tradition immediately diverts the question about her own death from the researcher to her religion and what she has been taught about what happens after death. Although she now finds herself in a rather secularized society, working close to death reminds her of what her denomination teaches. She expresses what is popularly called “Catholic guilt” in perceiving she “has to live better or become better” in order to “come to heaven to God”.

Dora, a 29-year-old woman from Southeast Asia identifying as a Roman Catholic, says some of the same as Charlotte but without guilt. Also Dora has experienced a change in her religious practices after having moved to Norway:

My family is very involved with religion. Every Sunday we had to attend mass. [. . .] I still believe in God. [. . .] but I do not need to go to church. God likes human beings and wants people to be good. I want to do it my way. [. . .] My mother looked after my child previously and always took my child to church. Now my child says: “Mum, you must pray to God before you go to sleep,” and such things. I do not have problems with this as long as nobody forces me to go to church, as long as no one can push me to do things in a certain way. I do it my way.

In her new context, Dora seems self-confident in that she has freed herself from the normative social obligations of religion such as having to attend services on a regular basis.

## Discussion

The intersection between religiosity, nursing, and health is well established (Page et al., 2020, p. 95), but the interconnectedness can be complex and intertwined with sociocultural concerns. How can we interpret the heterogeneous findings from this study?

Let us first try to understand our findings within the larger *societal framework*. Our findings thematize the relationship between the religious versus the secularized. Secularization is characterized by the three already mentioned features: differentiation (separation of state and church), diminishing religious faith and practices (decreased regular participation in religious services and ceremonial rituals), as well as the increasing “privateness” of religious practice (Taule, 2014, p. 10). Alongside with the secularization, globalized migration takes the nation’s populace in a more multireligious direction.

For the migrants who have moved to Norway from countries that are not as secularized as Norway, our findings indicate that religion is related to by the health care workers in various ways. In our interpretation, Isabella uses her own internalized religion as a sense of self-relief when she finds herself in the tension between the religious and the secularized. In East Africa, religion plays an important role both on a societal and on an individual level, and in Norway meaning-making has been detached from religious norms and authorities. It is within these multidirectional streams that Isabella is expected to find her feet.^10^ Grace and Eva are aware that the workplace does not rely on religion as an explanation or a resource in difficult situations, but nevertheless they do not hesitate to draw on their own religious resources (e.g., singing hymns and reading from the Bible) in their encounters with the patients. In our interpretation they do this as a means to bond with the patient’s spiritual life by creating a space of cross-cultural sharing and gathering (Green, 2009, p. 88; Taule, 2014, p. 12), in a mutually fulfilling exchange. In Odette’s Roman Catholic background, religious rituals are an important part of a human being’s life cycle. When she experiences an apparent lack of interest in religion from the Norwegian patients’ side, three theological matters are accentuated: First, from the reformation on, rituals around death in the Lutheran tradition were stripped down to a minimum as a human being’s faith and efforts before death were understood as more important than what happened after death. In the Lutheran understanding, the bereaved were freed from helping the soul to eternal life—as was an important task of the bereaved in the Roman Catholic tradition (Oftestad & Aavitsland, 2019, pp. 31-33). Second, in the Lutheran tradition, there are two sacraments, Baptism and the Lord’s Supper, whereas in the Catholic tradition there are seven. The Anointing of the Sick is the sacrament for those “in danger of death from sickness or old age” (cf. “The Anointing of the Sick,” 2020). When Odette refers to religious rituals surrounding the dying in her country of origin, these are ordained by the Roman Catholic Church, and provided by the priest. The priest in the Lutheran denomination, on the other hand, has no specific sacrament to provide the dying person with, and the religious rituals around death are therefore not officially ordained but open to individual interpretation and execution although many receive the Lord’s Supper. Third, Luther’s teaching about the two regiments has contributed to gradually make faith part of the private and not the public sphere. What Charlotte and Dora say about allowing themselves to leave out Sunday services can be interpreted in the light of being influenced by the context in which they live, or to be freed from the normative social obligation of having to attend church on a regular basis, an enculturation that in turn appears to create some moral dilemma (guilt) when Charlotte is asked to reflect about her own death. Charlotte seems to be in a tension between the religious and the secularized in that she reveals not feeling secure about the life she lives. Despite different lines of development between the Roman Catholic and Lutheran traditions, the interviews remind us that ideas and practices surrounding death and dying in any society are strongly tied to religious, cultural, and political structures.

When narrowing in to the *institutional framework* and the health care worker’s general work description, our findings can be interpreted in light of the obligation health care workers have to provide for a patient’s physical, psycho-social, and spiritual well-being (cf. World Health Organization, 2020). In a secularized society, religion is characterized by an increasing “privateness” of religious practice. If religion as a topic is not systematically talked about in the multicultural nursing home staff group, health care workers can easily develop private practices in their care for the patients’ spiritual well-being. When health care workers are to meet the spiritual needs of patients, this requires they have knowledge about what spiritual needs consist of, and have the courage to enter into the field (Cooper et al., 2020; Doka, 2011, p. 108; Holmberg et al., 2019, p. 1730; Jackson et al., 2016, pp. 290-293; McSherry et al., 2020). This again requires an organizational support that facilitates spiritual awareness and religious literacy not only toward patients but also among health care workers (cf. The Ministry of Health and Care Services, 2017; Giske & Cone, 2015; Hawthorne & Gordon, 2020, pp. 150-153; Lindheim, 2020, p. 27):

To do so, employees in nursing homes need religious literacy that helps them engage intelligibly in conversations about religion. Religious literacy can be developed by using existing conversational spaces in the workplace. These conversational spaces can become venues for learning and sharing if everyday religion is brought into the workplace and into the conversational space. (Lindheim, 2020, p. 27)

Our study reveals that the health care workers are attentive to religious issues, and that they meet these both in themselves and in the patients in various dynamic ways. None of the participants have, however, been asked by the management if their own cultural and religious backgrounds can play a role in their work in the nursing home (cf. Munkejord & Tingvold, 2019, p. 230; Rogstad & Solbrække, 2012, on integration and the underestimated qualifications and competencies of migrant health care workers). Individual and organizational strategies in the area of spiritual well-being for patients and health care workers alike seem not to be subject to discussion within the framework of the institutions. The fact that Isabella prays for the patients in her heart and is not completely sure whether or not this is allowed underlines the lack of a continuous discussion of these matters at her workplace. The way our participants relate to religion at work is therefore based on individual preferences and internalized practices. In order to comply with the professional obligation of holistic care, that is, to provide for a patient’s physical, psycho-social, and spiritual well-being, there is a need for a more organized conversation about spirituality and religion in the face of death among staff (Hawthorne & Gordon, 2020, p. 152), as “[D]eath, after all, may be the ultimate spiritual journey” (Doka, 2011, p. 108). In our consideration, this needs to be institutionally initiated and systematized and should not be left as the responsibility of the individual health care workers to negotiate on their own, as a private matter. By offering more organized conversations, the institution may foster trust, safe space, and authenticity to address all types of spiritual issues at the end of life, as “[I]t is very hard for staff who want to participate in and provide spiritual care when this is not supported by the organization” (Jackson et al., 2016, p. 291). It is our view that health care workers of migrant backgrounds may have knowledge and competence about religion that are of great value in the nursing home setting. In our data, this was most apparent when the health care worker shared a similar religious outlook as the patient. However, different religious outlooks, such as Philippa’s in our data material, may also be of value when facing existential and spiritual matters in end-of-life care (Hjort, 2020; Jackson et al., 2016, p. 291), but in order to reap such benefits health care workers must feel supported in engaging in such sensitive topics (cf. Egede-Nissen et al., 2017, on general prerequisites for quality care in multicultural workplaces). When people are facing death, questions of spiritual and religious character can be raised (Fortuin et al., 2019). If attentive and aware to do so, health care workers coming from less secularized societies than the Norwegian can draw on their own backgrounds and bring in knowledge and expertise that are needed into the nursing home setting, regardless of their respective religious affiliations.

If we zoom in even more and look at how *health care workers* use religion, this practice can be understood on at least two levels. We have already noted that working with people who approach death can trigger thoughts and feelings of existential character in the professional (Ramvi & Lavik, 2019). Health care workers’ applications of religion when being confronted with difficult feelings around death can be interpreted in light of the idea of religious coping strategies or mechanisms developed by the field of Psychology of Religion. Over the past decades, this field has contributed knowledge about how religion can serve as a resource in peoples’ lives (Danbolt et al., 2014; Koenig et al., 2012; Page et al., 2020, pp. 91-95; Pargament, 1997). For some of the health care workers in the present study, the outcomes of spiritual care practices were therapeutic and emotionally satisfying (Hawthorne & Gordon, 2020, p. 148). A pertinent example of this is how, in our material, Isabella uses her own internalized religion as a coping strategy as she is shocked by how death is dealt with in the institutions of the Norwegian welfare system. For her, the religious coping is a way of “binding herself back together” (cf. the literal meaning of the word religion; Koenig et al., 2012, p. 605). Eva’s and Grace’s practices underline their preunderstanding that as much as religion is meaningful to them, so they presuppose that it can also be a source of comfort and strength for patients who are approaching death. Grace became aware of, what we with Gadamer’s (2011) terminology can call, her own “horizon of understanding” when she first came to Norway and discovered how many Norwegians do not believe in God, that is, have a different horizon of understanding from her own. When Odette is confused by the patients’ presumable lack of interest in religion, this can be interpreted in light of her horizon of understanding as a Roman Catholic where religious rituals surround a member of the church from cradle to grave. In all these ways of applying religion, the participants—consciously or unconsciously—use their own horizons of understanding in a dynamic way to comprehend and make sense of what they experience in their encounter with people who are approaching death (cf. Giske & Cone, 2015).

In addition to these levels of interpretation, which show a wide span of health care workers’ experiences of religion, the data material can also be understood on a more fundamental level. Health care workers—as all other human beings—are confronted with death as “a merciless constant in human life” (Hviid Jacobsen, 2016, p. 17). Although humankind’s conception of death changes throughout history, and although death can evoke different feelings in individuals, this constant of death seems never to weaken its sting in human lives:

It seems as if the ability to learn to live with death is still—and perhaps will remain—one of the most daunting and difficult tasks with which humanity has to deal. (Hviid Jacobsen, 2016, p. 17)

Health care workers in nursing homes are not an exception, and their various application of own religious resources in demanding situations can be interpreted in the light of coming to terms with death as “a merciless constant in human life.” In addition, in this situation of approaching death—which can be experienced as unfamiliar and frightening—patients and health care workers seem to meet on familiar common ground. Despite cultural and other differences, religion may serve as a means of cross-cultural sharing between patient and personnel, and this sharing has the potential of creating a feeling of familiarity in the midst of the unfamiliar, and can in turn play a role in enhancing the well-being of both patients and health care workers.

## Conclusion

This article contributes to the understanding of how migrant health care workers relate to religion in a variety of ways in their care for the totality of patients who are approaching death in Norwegian nursing homes. Approaching death can evoke questions of spiritual character in both patients and personnel. Within our sample of participants, religious and cultural competence and knowledge of migrant nursing home staff were not asked for by the management. Coming from less secularized societies than the Norwegian, and caring for dying people in an institutionalized society, our material shows that religion has diverse meanings and contents among health care workers—both at a personal level and in relation to the patients. It also shows that the way our participants relate to religion at work is based on individual preferences and internalized practices.

## Implications for Holistic Nursing

Holistic nursing is highly aware of the interconnectedness of biological, social, psychosocial, and spiritual aspects in a human being. Holistic nursing practices require reflection among the staff. To support health care workers in performing holistic nursing in end-of-life care, facilitation of organized reflection groups about religion in the work place is needed. This is vital in order to integrate and develop what can be labelled *religious literacy* into the multicultural nursing home setting.

There is need for more studies on how migrant health care workers relate to religion—both with regard to their own potential reference to religious dimensions and with regard to the patient’s potential spiritual needs when facing death. Having migrated to a secularized and institutionalized society such as the Norwegian and working with people who are approaching death in such a society can evoke questions and issues of religious character, and more research is welcomed on this topic.

## Notes

1 The term *migrant* is here defined as a person who lives in Norway but was born outside of Norway having two non-Norwegian parents and four non-Norwegian grandparents. This definition corresponds with the description of the Norwegian term “innvandrer,” as defined by Statistics Norway (https://www.ssb.no/befolkning/artikler-og-publikasjoner/slik-definerer-ssb-innvandrere).

2 According to such an understanding of the term *spiritual* (as an inherent quality of being human), persons who, for instance, experience a decline in cognitive functions will still remain spiritual creatures.

3 This development can be explained in various ways but is most likely influenced by the reformer Martin Luther’s concept of the two ways in which he understands the reign of God in the world, the so-called *Zwei-Reiche*, or *Zwei-Regimente-Lehre*, the doctrine of the two governments (the spiritual and the secular). In simple terms, Luther’s idea is that the church is governed through the spiritual regiment, and that society is governed through the secular regiment (cf. Regimentslæren, https://snl.no/regimentsl%C3%A6ren).

4 On an individual level, however, religion still plays an integral part in many people’s lives in Norway (Botvar, 2010, pp. 23-24). One illustration of this is pointed at by A. Gustavsson who has investigated gravestones and memorial internet websites with view to bereavement in Norway and Sweden in recent times. When he analyzes memorial internet websites after the sudden death of a youth, he notices that Norwegian teenagers tend to include God and Jesus—for criticism or comfort—in their messages more often than Swedish teenagers do, and suggests that “[i]n critical situations one is more likely to appeal to God in Norway than would appear to be the case in Sweden” (Gustavsson, 2015, p. 231).

5 “[. . .] there has been a clear tendency toward secularization among those born in Norway and who do not have migrant background, whereas other groups pull in the opposite direction” (our translation of “[. . .] det har skjedd en klar sekularisering blant dem som er født i Norge og ikke har innvandringsbakgrunn, mens andre grupper trekker i motsatt retning”: https://www.ssb.no/kultur-og-fritid/artikler-og-publikasjoner/norge-et-sekulaert-samfunn). Whether or not secularization also can be seen as a global tendency is discussable (Horsfjord et al., 2018, p. 283).

6 The Official Norwegian Report *På liv og død—palliasjon til alvorlig syke og døende* (Departementenes sikkerhets, 2017, p. 27, Chapter 4, “Verdier”) is anchored in hospice thinking when it states that the professional, interdisciplinary team should strive to relieve the patient from the “total pain” of “[. . .] physical and psychological symptoms, social, existental and spiritual problems [. . .]” (our translation into English).

7 In 2009, in a circular from The Ministry of Health and Care Services titled *Rett til egen tro- og livssynsutøvelse*, it is stated that the municipal health and care services have a duty to provide for a resident’s ability to live and perform in accordance with their own view of life.

8 The study was approved by the Norwegian Centre for Research Data (NSD) in August 2017 with Project No. 54891.

9 When it comes to the concept “fusion of horizons,” Gadamer (2011) is sometimes criticized for being too optimistic. As the Biblical scholar Beth Elness-Hanson (2017) states, While “fusion” implies a completed process, it is however apparent from Gadamer’s writing that he is well aware that a state of being attained or completed is not possible. He readily states that understanding is an on-going and never ending process (Gadamer 2011: 293-294; Porter & Robinson 2011: 86–87, 91). Perhaps the present continuous tense, which the word “fusing” would relay, would be a more descriptive representation in English. Because of the equity in the established phrase “fusion of horizons,” I will continue to use fusion. (p. 36) On the same grounds we use the terminology *fusion of horizons* but stress this as an ongoing and never-ending process.

10 “[. . .] Norge går i en multireligiøs retning, heller enn befinner seg i en tiltakende sekulariseringsprosess” [Norway goes in a multireligious direction rather than in an emerging secularization process] (https://www.ssb.no/kultur-og-fritid/artikler-og-publikasjoner/norge-et-sekulaert-samfunn).

## References

[bibr1-0898010120973544] Ådland A. K., et al (2020). Nursing home staffs’ emotional experience of being in a close relationship to a resident who died: An interpretative phenomenological study [Manuscript in preparation]. Faculty of Health Sciences, University of Stavanger.

[bibr2-0898010120973544] The Anointing of the Sick. (2020). In Wikipedia. https://en.wikipedia.org/wiki/Anointing_of_the_Sick_in_the_Catholic_Church

[bibr3-0898010120973544] Ariès P. (1974). Western attitudes toward death from the middle ages to the present. Johns Hopkins University Press.

[bibr4-0898010120973544] Botvar P. K. (2010). Endringer i nordmenns religiøse liv—teorier om religiøs endring [Changes in religious lives of Norwegians – theories about religious change]. In Botvar P. K. Schmidt U. (Eds.), Religion i dagens Norge—mellom sekularisering og sakralisering [Religion in today’s Norway – between secularization and sacralization] (pp. 11-24). Universitetsforlaget.

[bibr5-0898010120973544] Braun V. Clarke V. (2006). Using thematic analysis in psychology. Qualitative Research in Psychology, 3(2), 77-101. 10.1191/1478088706qp063oa

[bibr6-0898010120973544] Claus G. (2018). Innvandrere stod for 1 av 6 årsverk innen omsorg [Immigrants did one of six annual works in caring]. Statistics Norway. https://www.ssb.no/helse/artikler-og-publikasjoner/innvandrerne-sto-for-1-av-6-arsverk-innen-omsorg

[bibr7-0898010120973544] Cooper K. L. Chang E. Luck L. Dixon K. (2020). How nurses understand spirituality and spiritual care: A critical synthesis. Journal of Holistic Nursing, 38(1), 114-121. 10.1177/0898010119882153 31596165

[bibr8-0898010120973544] Danbolt L. J. Engedal L. G. Stifoss-Hanssen H. Hestad K. Lien L. (2014). Religionspsykologi [Psychology of Religion]. Gyldendal Akademisk.

[bibr9-0898010120973544] Doka K. J. (2011). Religion and spirituality: Assessment and intervention. Journal of Social Work in End-of-Life & Palliative Care, 7(1), 99-109. 10.1080/15524256.2011.548049 21391080

[bibr10-0898010120973544] Egede-Nissen V. Sellevold G. S. Jacobsen R. Sørlie V. (2017). Ethical and cultural striving: Lived experiences of minority nurses in dementia Care. Nursing Ethics, 24(6), 752-766. 10.1177/0969733015624489 26811401

[bibr11-0898010120973544] Elness-Hanson B. (2017). Bible & theology in Africa: Vol. 24. Generational curses in the Pentateuch: An American and Maasai intercultural analysis (Holter K., Ed.). Peter Lang.

[bibr12-0898010120973544] European Association for Palliative Care. (n.d.). Spiritual care. https://www.eapcnet.eu/eapc-groups/reference/spiritual-care

[bibr13-0898010120973544] Fortuin N. P. M. Schilderman J. B. A.M. Venbrux E. (2019). Religion and fear of death among older Dutch adults. Journal of Religion, Spirituality and Aging, 31(3), 236-254. 10.1080/15528030.2018.1446068

[bibr14-0898010120973544] Fouron G. E. Glick-Schiller N. (2001). The generation of identity: Redefining the second generation within a transnational social field. In Cordero-Guzmán H. R. Smith R. C. Grosfoguel R. (Eds.), Migration, transnationalization and race in a changing New York (pp. 58-86). Temple University Press.

[bibr15-0898010120973544] Gadamer H.-G. (2011). Truth and method (2nd rev. ed., Weinsheimer J. Marshall D. G., Trans.). Continuum.

[bibr16-0898010120973544] Geertz C. (2017). The interpretation of cultures: Selected essays (Foreword by Darnton R.). Basic Books.

[bibr17-0898010120973544] Giske T. Cone P. H. (2015). Discerning the healing path: How nurses assist patient spirituality in diverse health settings. Journal of Clinical Nursing, 24(19-20), 2926-2935. 10.1111/jocn.12907 26215560

[bibr18-0898010120973544] Green J. W. (2009). Cultural diversity, spirituality, and end-of-life care. Reflective Practice: Formation and Supervision in Ministry, 29, 74-90.

[bibr19-0898010120973544] Gustavsson A. (2015). Death, dying and bereavement in Norway and Sweden in recent times. Humanities, 4(2), 224-235. 10.3390/h4020224

[bibr20-0898010120973544] Hawthorne D. M. Gordon S. C. (2020). The invisibility of spiritual nursing care in clinical practice. Journal of Holistic Nursing, 38(1), 147-155. 10.1177/0898010119889704 31777306

[bibr21-0898010120973544] Hjort K. (2020). Spiritualitet og sjælesorg [Spirituality and pastoral counseling]. Klinisk sygepleje, 34(2), 122-132. 10.18261/issn.1903-2285-2020-02-05

[bibr22-0898010120973544] Høeg I. M. Pajari I. (2013). Introduction to the Nordic issue of *Mortality*. Mortality, 18(2), 109-115. 10.1080/13576275.2013.785505

[bibr23-0898010120973544] Holmberg B. Hellström I. Österlind J. (2019). End-of-life-care in a nursing home: Assistant nurses’ perspectives. Nursing Ethics, 26(6), 1721-1733. 10.1177/0969733018779199 29950147

[bibr24-0898010120973544] Horsfjord V. Kloster S. T. Lende G. Løland O. J. (2018). Global kristendom. En samtidshistorie [Global Christianity. A contemporary history]. Universitetsforlaget.

[bibr25-0898010120973544] Hviid Jacobsen M. (2016). “Spectacular death”—Proposing a new fifth phase to Philippe Ariès’s admirable history of death. Humanities, 5(19), 1-20. 10.3390/h5020019

[bibr26-0898010120973544] Jackson D. Doyle C. Capon H. Pringle E. (2016). Spirituality, spiritual need, and spiritual care in aged care: What the literature says. Journal of Religion, Spirituality & Aging, 28(4), 281-295. 10.1080/15528030.2016.1193097

[bibr27-0898010120973544] Jong J. (2014). Ernest Becker’s psychology of religion forty years on: A view from social cognitive psychology. Zygon, 49(4), 875-889. 10.1111/zygo.12127

[bibr28-0898010120973544] Kittay E. F. Jennings B. Wasunna A. A. (2005). Dependency, difference and the global ethic of longterm care. Journal of Political Philosophy, 13(4), 443-469. 10.1111/j.1467-9760.2005.00232.x

[bibr29-0898010120973544] Koenig H. G. King D. E. Carson B. V. (2012). Handbook of religion and health (2nd ed.). Oxford University Press.

[bibr30-0898010120973544] Lindheim T. (2020). Developing religious literacy through conversational spaces for religion in the workplace. Nordic Journal of Religion and Society, 33(1), 16-29. 10.18261/issn.1890-7008-2020-01-02

[bibr31-0898010120973544] Loftsdóttir K. Jensen L. (2016). Introduction: Nordic exceptionalism and the Nordic “others.” In Jensen L. Loftsdóttir K. (Eds.), Whiteness and postcolonialism in the Nordic region: Exceptionalism, migrant others and national identities (pp. 1-11). Routledge.

[bibr32-0898010120973544] Martinsen K. (2017). Fra diakonisse til robot [From deaconess to robot]. Klinisk sygepleje, 31(1), 20-33. 10.18261/issn.1903-2285-2017-01-03

[bibr33-0898010120973544] McSherry W. Ross L. Attard J. van Leeuwen R. Giske T. Kleiven T. Boughey A., & members of the EPICC Network. (2020). Preparing undergraduate nurses and midwives for spiritual care: Some developments in European education over the last decade. Journal for the Study of Spirituality, 10(1), 55-71. 10.1080/20440243.2020.1726053

[bibr34-0898010120973544] Medås K. M. Blystad A. Giske T. (2017). Åndelighet i psykisk helseomsorg: et sammensatt og vanskelig tema [Spirituality in mental health care: A complex and difficult topic]. Klinisk sygepleje, 31, 273-286. 10.18261/issn.1903-2285-2017-04-04

[bibr35-0898010120973544] The Ministry of Health and Care Services. (2017). På liv og død—palliasjon til alvorlig syke og døende (The Official Norwegian Report NOU 2017:16) [In life and death – palliation to critically ill and dying]. https://www.regjeringen.no/contentassets/ed91baf5d25945b1a0b096c0ce376930/no/pdfs/nou201720170016000dddpdfs.pdf

[bibr36-0898010120973544] The Ministry of Health and Care Services. (2009). Rett til egen tro-og livssynsutøvelse [The right to practice own religion and belief]. https://www.regjeringen.no/no/dokumenter/i-62009-rett-til-egen-tros–og-livssynsu/id587577/

[bibr37-0898010120973544] Munkejord M. C. (2016). “Jeg jobber med hjertet”—innvandreres erfaringer med å arbeide i helse- og omsorgssektoren. Tidsskrift for omsorgsforskning, 2(3), 232-240. 10.18261/issn.2387-5984-2016-03-09

[bibr38-0898010120973544] Munkejord M. C. Tingvold L. (2019). Staff perceptions of competence in a multicultural nursing home in Norway. Social Science & Medicine, 232, 230-237. 10.1016/j.socscimed.2019.04.023 31103966

[bibr39-0898010120973544] The Norwegian Directorate of Health. (2019). Nasjonalt handlingsprogram for palliasjon i kreftomsorgen [National guidelines for palliation within cancer care]. https://www.helsedirektoratet.no/retningslinjer/palliasjon-i-kreftomsorgen-handlingsprogram

[bibr40-0898010120973544] The Norwegian Institute of Public Health. (2018). Death cause register 2018. http://statistikkbank.fhi.no/dar/

[bibr41-0898010120973544] The Norwegian Research Ethics Committees. (2014). General guidelines for research ethics. https://www.forskningsetikk.no/en/guidelines/general-guidelines/

[bibr42-0898010120973544] Oftestad E. A. (2019). Kunsten å dø: eutanasi. [The art of dying: Euthanasia]. Vårt Land. http://www.verdidebatt.no/innlegg/11752162-kunsten-a-do-eutanasi

[bibr43-0898010120973544] Oftestad E. A. Aavitsland K. B. (2019). Kunsten å dø [The art of dying]. In Rasmussen T. (Ed.), Å minnes de døde: Døden og de døde i Norge etter reformasjonen [To bear in mind the dead: Death and the dead in Norway after the Reformation] (pp. 27-48). Cappelen Damm Akademisk.

[bibr44-0898010120973544] Oman D. Thoresen C. E. (2005). Do religion and spirituality influence health? In Paloutzian R. F. Park C. L. (Eds.), Handbook of the psychology of religion and spirituality (pp. 435-459). Guildford Press.

[bibr45-0898010120973544] Page R. L. Peltzer J. N. Burdette A. M. Hill T. D. (2020). Religiosity and health: A holistic biopsycosocial perspective. Journal of Holistic Nursing, 38(1), 89-101. 10.1177/0898010118783502 29957093

[bibr46-0898010120973544] Pargament K. I. (1997). The psychology of religion and coping: Theory, research, practice. Guilford Press.

[bibr47-0898010120973544] Pargament K. I. (2007). Spiritually integrated psychotherapy: Understanding and addressing the sacred. Guilford Press.

[bibr48-0898010120973544] Paul Victor C. G. Treschuk J. V. (2020). Critical literature review on the definition clarity of the concept of faith, religion, and spirituality. Journal of Holistic Nursing, 38(1), 107-113. 10.1177/0898010119895368 31858879

[bibr49-0898010120973544] Porter S. E. Robinson J. C. (2011). Hermeneutics: An introduction to interpretive theory. William B. Eerdmans.

[bibr50-0898010120973544] Ramvi E. Lavik M. H. (2019). ‘Jeg klarer ikke finne noen ord’. Å arbeide tett på døden [‘I cannot find the words’. Working close to death]. In Gripsrud B. H. Thoresen L. (Eds.), Alt som lever må dø. Døden som tverrfaglig kunnskapsfelt [All that lives must die. Death as an interdisciplinary field of knowledge] (pp. 33-54). Scandinavian Academic Press.

[bibr51-0898010120973544] Rogstad J. Solbrække K. N. (2012). Velmenende likegyldighet. Konflikt og integrasjon i et flerkulturelt sykehus [Benign indifference. Conflict and integration in a multicultural hospital]. Sosiologisk tidsskrift, 4(20), 315-338.

[bibr52-0898010120973544] Schmidt U. (2010a). Norge: et religiøst pluralistisk samfunn? [Norway: A religious pluralistic society?] In Botvar P. K. Schmidt U. (Eds.), Religion i dagens Norge—mellom sekularisering og sakralisering [Religion in today’s Norway – between secularization and sacralization] (pp. 25-43). Universitetsforlaget.

[bibr53-0898010120973544] Schmidt U. (2010b). Religion i dagens Norge: sekularisert? privatisert? pluralisert? [Religion in today’s Norway: Secularized? Privatized? Pluralized?] In Botvar P. K. Schmidt U. (Eds.), Religion i dagens Norge—mellom sekularisering og sakralisering [Religion in today’s Norway – between secularization and sacralization] (pp. 196-204). Universitetsforlaget.

[bibr54-0898010120973544] Spirituality. (2020). In Wikipedia. https://en.wikipedia.org/wiki/Spirituality

[bibr55-0898010120973544] Sørensen A. S. Høystad O. M. Bjurström E. Vike H. (2008). Nye kulturstudier: en innføring [New Cultural Studies: An introduction]. Scandinavia Academic Press.

[bibr56-0898010120973544] Taule L. (2014). Norge—et sekulært samfunn? Samfunnsspeilet, 1, 9-16. https://www.ssb.no/kultur-og-fritid/artikler-og-publikasjoner/norge-et-sekulaert-samfunn

[bibr57-0898010120973544] Thoresen L. (2017). Døden på nordiske sykehjem—medikalisert? [Death in Nordic nursing homes – medicalized?] In Bondevik H. Madsen O. J. Solbrække K. N. (Eds.), Snart er vi alle pasienter. Medikalisering i Norden [Soon we are all patients. Medicalization in the Nordic countries] (pp. 263-287). Scandinavian Academic Press.

[bibr58-0898010120973544] Tingvold L. Fagertun A. (2020). Between privileged and oppressed? Immigrant labor trajectories in Norwegian long-term care. Sustainability, 12(11), 4777. 10.3390/su12114777

[bibr59-0898010120973544] The United Nations. (1948). Universal declaration on human rights. https://www.un.org/en/universal-declaration-human-rights/

[bibr60-0898010120973544] The United Nations. (2017). International migration report 2017 [Highlights]. https://www.un.org/en/development/desa/population/migration/publications/migrationreport/docs/MigrationReport2017_Highlights.pdf

[bibr61-0898010120973544] Walter T. (2012). Why different countries manage death differently: A comparative analysis of modern urban societies. British Journal of Sociology, 63(1), 123-145. 10.1111/j.1468-4446.2011.01396.x 22404392

[bibr62-0898010120973544] Wojtkowiak J. (2018). Towards a psychology of ritual: A theoretical framework of ritual transformation in a globalising world. Culture & Psychology, 24(4), 460-476. 10.1177/1354067X18763797

[bibr63-0898010120973544] The World Health Organization. (2020). Majority provider of palliative and end-of-life care services in community for dementia [public, private]. https://www.who.int/data/gho/indicator-metadata-registry/imr-details/5109

